# Hide, Keep Quiet, and Keep Low: Properties That Make *Aspergillus fumigatus* a Successful Lung Pathogen

**DOI:** 10.3389/fmicb.2016.00438

**Published:** 2016-04-06

**Authors:** Natalia Escobar, Soledad R. Ordonez, Han A. B. Wösten, Pieter-Jan A. Haas, Hans de Cock, Henk P. Haagsman

**Affiliations:** ^1^Department of Microbiology, Institute of Biomembranes, Utrecht UniversityUtrecht, Netherlands; ^2^Department of Infectious Diseases and Immunology, Division Molecular Host Defence, Utrecht UniversityUtrecht, Netherlands; ^3^Department of Medical Microbiology, University Medical Center UtrechtUtrecht, Netherlands

**Keywords:** *Aspergillus fumigatus*, *Aspergillus niger*, infection, lung, hyphal growth, internalization, epithelial cells, germination

## Abstract

Representatives of the genus *Aspergillus* are opportunistic fungal pathogens. Their conidia can reach the alveoli by inhalation and can give rise to infections in immunocompromised individuals. *Aspergillus fumigatus* is the causal agent of invasive aspergillosis in nearly 90% of the cases. It is not yet well-established what makes this fungus more pathogenic than other aspergilli such as *A. niger*. Here, we show that *A. fumigatus* and *A. niger* conidia adhere with similar efficiency to lung epithelial A549 cells but *A. fumigatus* conidia internalized 17% more efficiently. Conidia of both aspergilli were taken up in phagolysosomes 8 h after the challenge. These organelles only acidified in the case of *A. niger*, which is probably due to the type of melanin coating of the conidia. Viability of both types of conidia was not affected after uptake in the phagolysosomes. Germination of *A. fumigatus* and *A. niger* conidia in the presence of epithelial cells was delayed when compared to conidia in the medium. However, germination of *A. niger* conidia was still higher than that of *A. fumigatus* 10 h after exposure to A549 cells. Remarkably, *A. fumigatus* hyphae grew mainly parallel to the epithelium, while growth direction of *A. niger* hyphae was predominantly perpendicular to the plane of the cells. Neutrophils reduced germination and hyphal growth of *A. niger*, but not of *A fumigatus*, in presence of epithelial cells. Taken together, efficient internalization, delayed germination, and hyphal growth parallel to the epithelium gives a new insight into what could be the causes for the success of *A. fumigatus* compared to *A. niger* as an opportunistic pathogen in the lung.

## Introduction

*Aspergillus* species have a global distribution and are among the most abundant fungi (Gugnani, 2003; Machida and Gomi, 2010; Shams-Ghahfarokhi et al., 2014). They mainly feed on dead organic material but are also opportunistic pathogens of plants, animals, and humans (Krijgsheld et al., 2013). An *Aspergillus* colony can form a few billion conidia that are dispersed by wind, water droplets, and insects. Humans inhale daily 200–300 of these asexual reproductive structures of *Aspergillus fumigatus* alone (Mullins et al., 1984; Kasprzyk, 2008). *Aspergillus* infections rarely occur in immuno-competent individuals, indicating efficient clearance of conidia by pulmonary defense mechanisms (Hope, 2009). However, infections occur as a consequence of suppressed or impaired host immunity (Rüping et al., 2008; Henriet et al., 2013). Inhaled conidia that reach the alveoli pose a significant threat for these patients. Conidia may internalize within the lung epithelium, while the hyphae that result from their germination may cross the alveolar epithelium to cause invasive aspergillosis (IA). IA is accompanied by long-term hospitalization, intensive antifungal therapies, and high rates of mortality (Morgan et al., 2005; Cornillet et al., 2006).

The integrity of the epithelium in the upper and lower respiratory tract and the proper functioning of ciliated epithelium are essential for clearance of conidia and prevention of infection. Alveolar macrophages, other surveying immune cells such as neutrophils, defense components, and lung surfactant play a crucial role as well (Luther et al., 2008; Brown, 2011; Hasenberg et al., 2011). The role of neutrophils in defense against *A. fumigatus* has been relatively well-studied (Mircescu et al., 2009). Neutrophils phagocytose resting and swollen conidia as well as conidia with short germ tubes. In addition, they restrict hyphal growth and dissemination by forming neutrophil extracellular traps (NETs; McCormick et al., 2010a; Bianchi et al., 2011). This web-like structure is produced as a result of neutrophil induced apoptosis and consists of DNA, chromatin and cytoplasmic and granular proteins (Brinkmann et al., 2004). However, conidia restrict recognition by neutrophils by their rodlet and melanin layers that coat them (Bruns et al., 2010; Chai et al., 2010; Amin et al., 2014).

*Aspergillus fumigatus* is the main cause of IA and allergic aspergillosis (Stevens et al., 2003; Chakrabarti et al., 2011). It is often isolated from patients presenting symptoms, even though in the majority of the cases cultures remain negative (Janssen et al., 2011). Aspergillosis can also be caused by other *Aspergillus* species such as *A. flavus, A. niger*, and *A. terreus* (Schuster et al., 2002; Steinbach et al., 2004; Saracli et al., 2007; Bukhari and Alrabiaah, 2009; Ozhak-Baysan et al., 2010; Bathoorn et al., 2013). However, the reason why *A. fumigatus* is such a prominent opportunistic pathogen is not yet understood. Conidial size, cell wall composition, and secretion of secondary metabolites play an important role in *Aspergillus* infection (Pel et al., 2007; Sugui et al., 2007; Braaksma and Punt, 2008; Tiralongo et al., 2009; Thywissen et al., 2011; Kwon-Chung and Sugui, 2013). The relatively small diameter of its conidia (2–3 μm) would make *A. fumigatus* a more potent pathogen when compared to other *Aspergillus* species. A small conidial size favors deposition at the alveolar surface and internalization by phagocytic and non-phagocytic cells (McCormick et al., 2010b). Conidia from *A. niger* and *A. flavus* that have a diameter of 4–6 μm have a lower probability to reach the alveoli, and are therefore mainly cleared in the conductive airways. Nevertheless, conidia of *A. terreus* have a similar size (2–4 μm) as those of *A. fumigatus* but do not cause infections as frequently as *A fumigatus*, indicating that also other factors contribute to *A. fumigatus* infections (Deak et al., 2009). Indeed, *A. fumigatus* conidia but not those of *A. terreus* prevent acidification of phagolysosomes in macrophages (Slesiona et al., 2012). This was attributed to differences in the structure of melanin that is formed by these aspergilli. *A. niger* conidia form another type of melanin than *A. fumigatus.* The former synthesize melanin via L-3,4-dihyroxyphenylalanine (L-DOPA) and 5,6-dihydroxynindole, while the latter makes use of 1,8 dihydroxynaphtalene to form DHN melanin (Eisenman and Casadevall, 2012; Pal et al., 2014).

In this study we have compared adhesion, internalization, germination, and outgrowth of conidia of *A. fumigatus* and *A. niger* using an alveolar epithelium model. We hypothesize that the comparison between these two species, presenting different morphological and secretory characteristics, will point out to some of the features that make *A. fumigatus* a more successful pathogen. Interestingly, results showed higher internalization efficiency and delayed germination, for *A. fumigatus* compared to *A. niger*. At a later stage of infection hyphal growth direction differed between these two species which might affect efficient recognition of *A. fumigatus* by neutrophils. Overall these observations show how *A. fumigatus* could cause a more successful infection than *A. niger*.

## Materials and Methods

### Strains and Growth Conditions

The strains selected for *A. niger* and *A. fumigatus* have been studied extensively (**Table 1**). For *A. fumigatus* the strain A293 has been used for assessing internalization and infection in A549 cells (Wasylnka and Moore, 2003). This strain originates from a clinical isolate (Pain et al., 2004; Nierman et al., 2005), which makes it more representative for this specific study. For *A. niger* no experiments involving infection and interaction with epithelial cells have been described to our knowledge. Therefore, the strains used are environmental strains, completely sequenced and often used in fungal biology (Yuan et al., 2008). *A. niger* AV112d.7 expressing dTomato is a derivate of *A. niger* CB-A112.11T which is derived from *A. niger* NW249. NW249 is a derivative of *A. niger* N402 as described by Jalving et al. (2000). AV112d.7 carries multiple copies of the transforming construct. Single integrations did not result in a fluorescence strong enough for fluorescence microscopy. Therefore, two representative strains of each transformation were selected for further analysis and characterization. Growth and sporulation of these transformants were not affected when compared to the parental strain. Although unlikely, we cannot exclude the possibility that the fluorescent derivative of *A. niger* strain used in this study might differ in some aspects to their non-fluorescent parent.

**Table 1:** Strains used in this study.

Strains	Construct	Parental strain	Description of strain
*Aspergillus fumigatus*			
AF 293			Wild type (Pain et al., 2004)
AF 293.1^a^	pRG3AMA1-RFP	AF293	Strain expressing RFP (Leal et al., 2010)
AF Δ*pksP*^b^		CEA17	Non-melanised mutant, partial deletion of *pksP* (Amin et al., 2014)
*A. niger*			
N402		NRRL3	Short conidiophore mutant (Bos et al., 1988)
AV112d.7^c^	PglaA:dTomato	CB-A112.11	Strain expressing *dtomato* (Vinck et al., 2011)
JP1.1	*pptA::AopyrG*	AB4.1	Non-melansied mutant, deletion of *pptA* (Jørgensen et al., 2011)

*^a^Provided by Michelle Momany (University of Athens, Athens, GA, USA).*

*^b^Provided by Axel A. Brakhage [Leibniz Institute for Natural Product Research and Infection Biology – Hans Knöll Institute (HKI), Jena, Germany].*

*^c^AV112d.7 is derived from a similar strain as the one used as a wild type in this study (N402). The paper previously published by our lab (Vinck et al., 2011) states that this strain behaves similarly as N402.*

Spores were grown on PDA agar plates for 3 days at 37°C. Conidia were harvested with 0.85% (w/v) NaCl and filtered through three layers of miracloth (Merck Millipore Corporation, Darmstadt, Germany) to remove pieces of mycelium. Suspensions were adjusted to 10^8^ conidia/ml after counting the conidia with a Bürker chamber.

### Cell Cultures and Fungal Infection

Cells of the human lung carcinoma epithelial cell line A549 (ATCC, CCL-185) were maintained by serial passage in dulbecco’s modified eagle medium (DMEM) culture medium (Ref. code: 41966-029 GIBCO, Life Technologies, Paisley, UK) with 10% (v/v) fetal calf serum (FCS; Bodinco BV, Alkmaar, the Netherlands). A549 cells were seeded at a concentration of 2 × 10^5^ cells/ml, and cultured at 37°C and 5% CO_2_ until a confluent monolayer was formed. Cells were challenged with 2 × 10^6^ fungal conidia/well, in 48-well plates (Corning^®^, Costar^®^, New York, USA) suspended in DMEM + 10% FCS. This concentration of conidia was previously tested in order to achieve a measurable infection in A549 cells and is similar to the concentration used in previous studies such as the one by Wasylnka and Moore (2003). For experiments that required fixation prior to fluorescence microscopy, A549 were cultured in 48-well culture plates, containing 8-mm glass coverslips (WPI international BV, Europe). For live imaging and for experiments requiring the maintenance of hyphal growth directionality, cells were grown in μ-slide eight well glass bottom chambers (Ibidi^®^, Munich, Germany).

### Association and Internalization of Conidia upon Interaction with A549 Cells

Prior experiments, A549 cells were grown on 8-mm glass coverslips (WPI international BV, Europe). Conidia were added to A549 cells and incubated for 4 h. Unbound conidia were removed carefully by washing three times with DMEM (pre-warmed to 37°C). Adhering conidia were visualized with 0.003% Calcofluor-white (CFW; SIGMA-ALDRICH, Buchs, Switzerland). The dye was added for 5 min at 37°C followed by two washing steps with pre-warmed DMEM. Cells were fixed with 4% paraformaldehyde (PFA; SIGMA-ALDRICH, Buchs, Switzerland) for 5 min at 4°C and 20 min at room temperature. PFA background fluorescence was quenched by incubation with 20 mM NH_4_Cl (Merck Millipore, Darmstadt, Germany) for 20 min at RT. Coverslips were mounted with FluorSave^TM^ (Merck Millipore, Darmstadt Germany) onto glass slides. To determine the total number of conidia associated with the lung epithelium, 12 fields at the coverslip were randomly chosen. Hoechst (Life Technologies, Paisley, UK) stain was used to count the total number of cells per field.

Association values are expressed as the percentage of total conidia that had bound to the A549 cells. Obtained values were an average from five separate experiments. At least 100 conidia were counted per strain in all experiments. For evaluating conidial internalization, Z-stacks of 10 randomly chosen sites were made. Four separate experiments using at least 200 conidia per strain were included in the analysis. Conidia stained with CFW were counted as non-internalized, while conidia that only showed mRFP or dTomato fluorescence were counted as internalized conidia. For conidial internalization values are presented either as the percentage of internalized conidia from the total number used for the challenge, or the total number of associated conidia. Analysis of images is described in the confocal microscopy section below. Viability of A549 internalized conidia was measured using a nystatin protection assay (Wasylnka and Moore, 2002). Briefly, conidia were added to A549 cell and incubated for 4 h to allow internalization. Cells were washed two times with DPBS and incubated with 100 μg/mL of nystatin. Incubation was followed for 3 h and cells were washed two times. Finally, cells were detached from well plates using 0.05% trypsin and serial dilutions were plate on PDA agar. Conidia were count after 3 days of incubation at 37°C.

### Germination and Directionality of Hyphal Growth upon Interaction with A549 Cells

For germination experiments A549 cells were grown on 8-mm glass coverslips (WPI international BV, Europe) and in μ-slide eight well chambers (Ibidi^®^, Munich, Germany) for observing hyphal growth directionality. CFW was used to visualize conidia and hyphae outside the A549 cells. After 6 h of infection, samples were followed every hour in order to track germinating conidia. As a control for germination, conidia alone were grown in the same cell culture medium used for A549 cells at 37°C, 5% CO_2_. Observations were made every hour until hyphal length reached a size comparable to the observed in the presence of A549 cells. Three separate experiments were included in the analysis, using at least 100 conidia per experiment.

To assess hyphal growth directionality, hyphae from both strains were grown until they reached similar lengths. The latter times for observation were taken from germination experiments were we saw a lag in growth for *A. fumigatus* compared to *A. niger*. For epithelial visualization, A549 cells were stained with Hoechst (1 μg/ml; Life Technologies, Paisley, UK). Samples were fixed with PFA (see above) and visualized by confocal microscopy. For hyphal directionality, Z-stacks covering the region between the bottom of the A549 layer until the tips of external hyphae were made. Measurements in the Y and X plane were analyzed. Three biological replicas were analyzed using approximately 10 fields per slide per experiment The ratio of hyphae growing perpendicularly vs. those growing in parallel and perpendicularly to the epithelium was also calculated. This was done by counting more than 50 hyphae per strain from two independent experiments.

### Internalization Blockers

Cytochalasin B (10 μM; SIGMA-ALDRICH, Zwijndrecht, The Netherlands) and/or 20 μM nocodazole (SIGMA-ALDRICH, Zwijndrecht, The Netherlands) were used to block internalization pathways. These concentrations were obtained from cytotoxicity experiments with A549 cells together with the comparison with other article (Bose et al., 2001). A549 cells were treated with the blockers for 30 min prior to infection with conidia. To evaluate treatments, 10 fields per slide were chosen from two separate experiments using >600 conidia. For analysis, each conidium was scored as either inside or outside the epithelial cells. Chi-square proportion test was performed using a *z*-test (α = 0.01) and adjusting *P*-values for multiple comparisons using the Bonferroni correction method.

### Localization of Conidia in Phagolysosomes and Their Acidification

Antibody LAMP-1-FITC (BD Transduction Laboratories) was used to detect co-localized conidia with phagolysosomes 2 and 8 h after the challenge. Cells were fixed as described above and permeabilized with 0.1% saponin in dulbecco’s phosphate buffered saline (DPBS; Ref. code 14190-094 SIGMA-ALDRICH, Zwijndrecht, The Netherlands) containing 20 mM NH_4_Cl and 2% bovine serum albumin (BSA; SIGMA-ALDRICH, Zwijndrecht, The Netherlands). Cells were incubated with 100-fold diluted LAMP-1 antibodies for 45 min at RT. Slides were washed once with PBS and mounted with FluoroSave. Percentages of colocalization were calculated by analyzing at least 50 conidia in each of three separate experiments.

Acidification of lysosomes was evaluated using LysoSensor^TM^ Green DND-189 (Life Technologies, Eugene, OR, USA) and the pH sensitive fluorophore CypHer5E-NHS Ester (CypHer5E; GE Healthcare, Bilthoven, Netherlands). A549 cells were incubated for 45 min with 50 nM LysoSensor^TM^. For labeling with CypHer5E, 200 μL conidia suspension (10^8^ conidia/ml) was incubated at RT for 2 h with 6 μL 10 mg/ml CypHer5E in 0.5 M sodium carbonate buffer, pH 8.3. Conidia were washed twice with this buffer with intermediate centrifugation for 5 min at 10.000 rpm. In order to relate CypHer5E fluorescence with pH, a calibration curve of fluorescence was used made with 0.5 M PBS (pH 7) and mixtures of 0.1 M citric acid and 0.2 M di-sodium phosphate buffer (2:1, 1:1, and 1:2 for pH 4, 5, and 6, respectively). Analyzed data was obtained from three separate experiment counting at least 100 conidia per condition.

### Epithelial Cell Infections with *A. fumigatus* and *A. niger* in the Presence of Neutrophils

Human polymorphonuclear neutrophils (PMNs) were isolated from whole blood of healthy donors following the Histopaque-Ficoll gradient protocol as previously described (Bestebroer et al., 2007). Informed consent was obtained from all subjects and was provided according to the Declaration of Helsinki. Approval was obtained from the medical ethics committee of the University Medical Center Utrecht (Utrecht, The Netherlands). PMNs (2 × 10^6^ cells/well) were incubated with A549 cells for 3 h at the moment conidia had started to form germ tubes. This concentration of neutrophils was previously tested in order to have a measurable amount of neutrophils at A549 surface at the moment of measurements. As a reference, conidia from both strains were grown without epithelial cells and treated with PMNs right after germination. Data was taken from three separate experiments, by observing at least 100 conidia per condition.

### Confocal Microscopy

Confocal Images were acquired with a Leica SPE-II using the 63x ACS APO (NA = 1.3) or 40x PLAN APO (NA = 1.25–0.75) objectives. Imaging was performed using a quadruple band beam splitter for the 405, 488, 561, and 647 nm laser lines. Identical settings were used when comparing fluorescence intensities between samples. Fluorescence emission of CFW was detected using the spectral band 460–480 nm. Red fluorescence emission of mRFP, dTomato, and CypHer5E was detected using spectral bands of 600–650, 562–600, and 650–690 nm, respectively, while LAMP-1-FITC and LysoSensor^TM^ fluorescence emission was detected with the 490–520 nm spectral band. For the analysis and processing of images the Fiji image processing package of ImageJ (www.fiji.sc) was used.

### Statistical Analysis

GraphPad Prism Software (GraphPad Software, Inc., La Jolla, CA, USA) was used for statistical analysis. Differences were analyzed using the student’s unpaired test, (two-tailed *P*-value) or one-way ANOVA. *P*-values of ≤0.05 were considered significant and ≤0.001 as highly significant. Analysis of internalization and internalization blockers values was performed using IBM SPPS Statistics for Windows, Version 22.0. Values where scored as in or out and treated as binary data. Pearson Chi-square test was used evaluating significance with *P*-values ≤ 0.05.

## Results

### Association and Internalization of Conidia by A549 Cells

A monolayer of A549 cells was incubated with *A. fumigatus* and *A. niger* conidia. Association and internalization of the conidia was assessed up to 4 h after the challenge. Adherence of the *A. fumigatus* and *A. niger* conidia to A549 cells was not significantly different. It reached its maximum within 2 h where 1% of both *A. fumigatus* and *A. niger* conidia bound to the A459 cells (**Supplementary Figure S1**). Internalization of *A. fumigatus* and *A. niger* reached its maximum after 4 h, with 84 and 67%, respectively. Statistically, *A. fumigatus* internalization was 1.2-fold more efficient when compared to *A. niger*.

**FIGURE 1:**
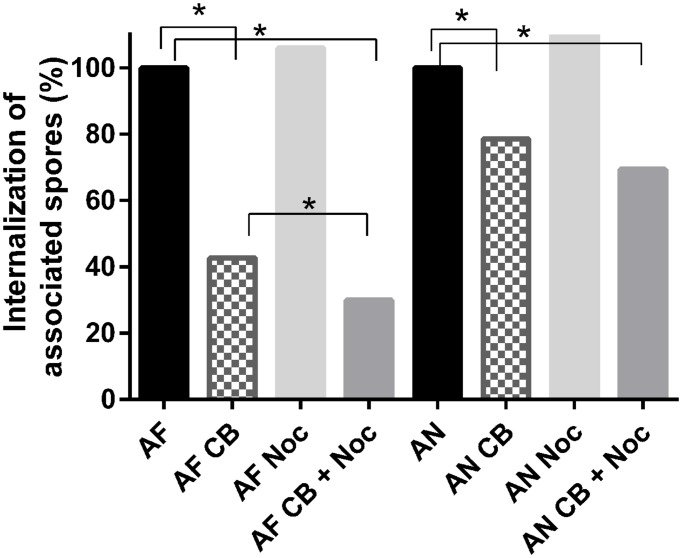
**Cytochalasin-B and nocodazole block internalization of *Aspergillus fumigatus* more effectively than that of *A. niger*.** Internalization of *A. niger* (AN) and *A. fumigatus* (AF) by A549 after treatment with 10 μM cytochalasin-B (CB), and/or 20 μM nocodazole (Noc). For analysis, each conidium was scored as either inside or outside the epithelial cells. A chi-square proportion test was performed using a *z*-test (α = 0.01) and adjusting *P*-values for multiple comparisons using the Bonferroni correction method.^∗^ Indicates significant difference.

Cytochalasin-b and nocodazole were used to investigate the role of actin and microtubules during internalization of conidia in A549 cells. These blockers induce actin disruption and interfere with microtubule formation, respectively (Ellinger et al., 2001; Wasylnka and Moore, 2002; Ibrahim-Granet et al., 2003). Treatment of epithelial cells with 10 μM of cytochalasin-b inhibited internalization of conidia by 57 and 21% in the case of *A. fumigatus* and *A. niger*, respectively. For *A. fumigatus* the treatment using both cytochalasin-b and nocodazole resulted in an increased inhibition compared to cytochalasin-b alone, decreasing internalization from 50 to 70%. For *A. niger* the addition of (20 μM) nocodazole did not significantly affect cytochalasin-b activity changing internalization from 31 to 44% (**Figure 1**).

### Localization of Conidia at Phagolysosomes and Their Acidification

**FIGURE 2:**
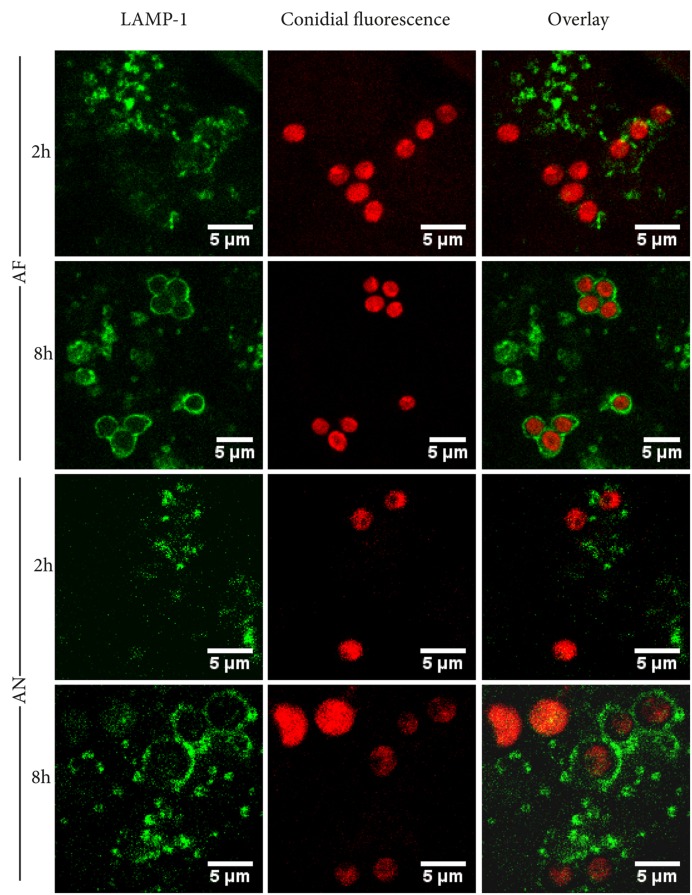
***Aspergillus fumigatus* and *A. niger* conidia co-localize with LAMP-1 after 8 h of infection.** Representative pictures of RFP or dTomato labelld *A. niger* and *A. fumigatus* conidia, co-localizing with FITC-labeled phagolysosomal marker LAMP-1 after 2 and 8 h of incubation. Images shown are representative of one of three separate experiments.

The lysosomal-associated membrane protein 1 (LAMP-1) was used as a marker to determine whether internalized conidia were located inside phagolysosomes. After 2 h of incubation, 29 and 21% of the *A. fumigatus* and *A. niger* conidia, respectively, were co-localized with LAMP-1. These numbers increased to 59 and 54% after 8 h of incubation (**Figure 2**).

**FIGURE 3:**
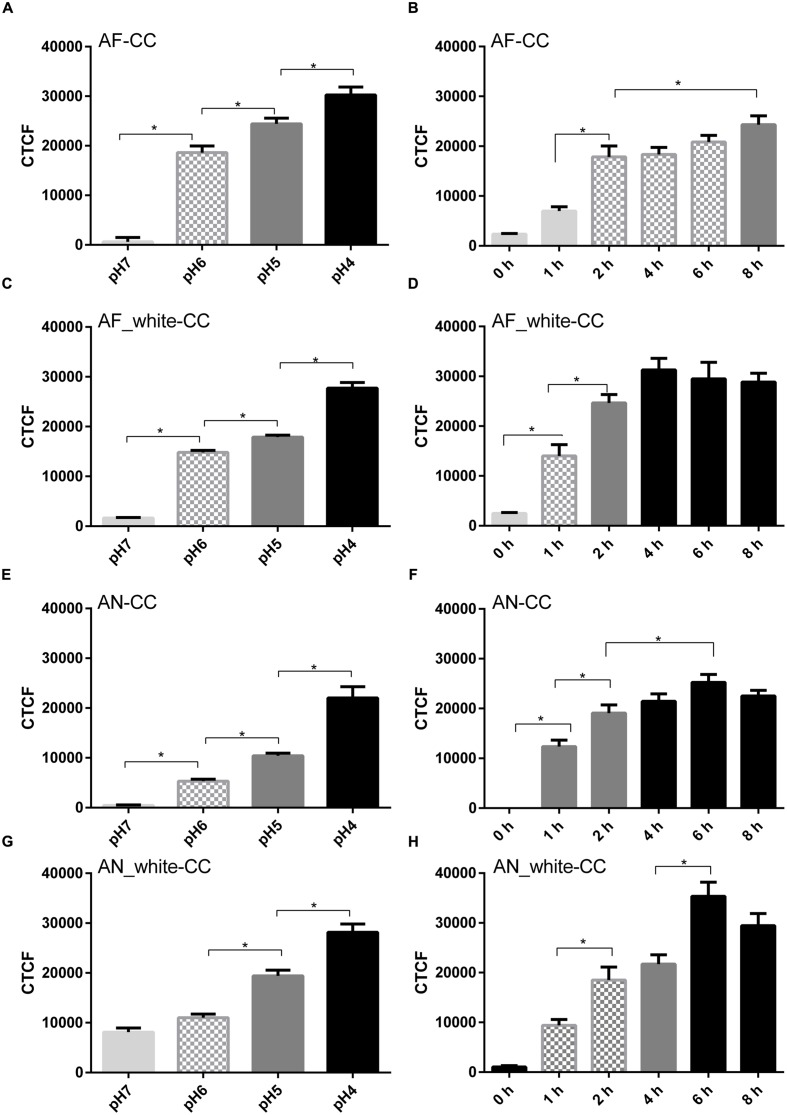
***Aspergillus fumigatus* melanin inhibits acidification of cellular compartments.** Tracking of acidification inside cellular compartments containing Cy-pHer5e-labeled conidia (CC). Wild-type *A. niger* and *A. fumigatus* and of white strains (AN_white and AF_white). pH calibration curve of AF-CC **(A)**, AF_white-CC **(C)**, AN-CC **(E)**, and AN_white-CC **(G)** and total fluorescence in time of AF-CC **(B)**, AF_white-CC **(D)**, AN-CC **(F)**, and AN_white-CC **(H)**. Values are expressed as the mean of the corrected total fluorescence (CTCF). Bars represent standard error of the mean. ^∗^Indicates significant difference. Data obtained from three separate experiments analyzing at least 100 conidia per condition were analyzed.

Labeling of acidic compartments by LysoSensor^TM^ and labeling of conidia by a pH sensitive dye (CypHer5E) were used to address whether acidification of the phagolysosomes occurred. LysoSensor strongly stained compartments in A549 cells. However, internalized conidia of both *A. fumigatus* and *A. niger* were not surrounded by the label, even 8 h after the challenge (**Supplementary Figure S2**). To confirm the LysoSensor results, conidia were labeled with the pH sensitive dye CypHer5E. Fluorescence of CypHer5E bound to *A. fumigatus* conidia indicated a pH of 6 between 2 and 6 h after the challenge and a pH of 5 after 8 h (**Figures 3A,B**). In contrast, fluorescence of CypHer5E bound to *A. niger* conidia showed a gradual drop to pH 4 after 4 h, remaining stable until 8 h after the challenge (**Figures 3E,F**).

It has been shown that DHN-melanin of *A. fumigatus* conidia inhibit phagolysosome acidification (Jahn et al., 2002; Thywissen et al., 2011). To test whether *A. niger* melanin also inhibits the acidification process, conidia of white strains of *A. fumigatus* and *A. niger* were used in CypHer5E experiments. Conidia of the white strain of *A. fumigatus* resulted in phagolysosome acidification, reaching pH 4 after 4 h of challenge (**Figures 3C,D**). In contrast, both white and wild-type strains of *A. niger* did not decrease acidification and phagolysosomes reached pH 4 after 4 and 6 h, respectively (**Figures 3E–H**). These experiments show that melanin coating of *A. niger* conidia does not impact acidification in phagolysosomes while *A. fumigatus* melanin coat does.

### Germination and Hyphal Growth Directionality

**FIGURE 4:**
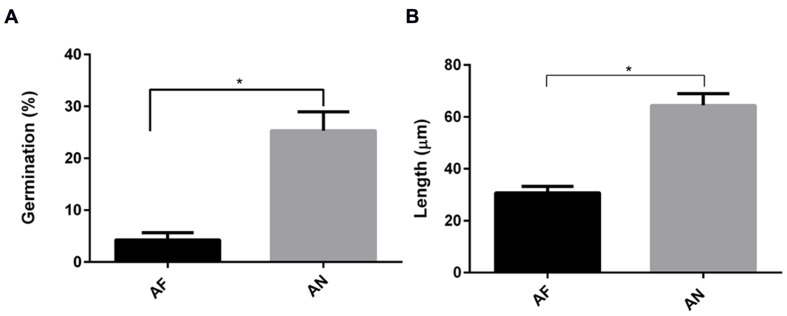
**Germination and hyphal length of *A. fumigatus* are more effectively decreased in the presence of A549 cells than that of *A. niger*. (A)** Germination and **(B)** hyphal length. Bar represents standard error of the mean. ^∗^ Indicates significant difference. Data are obtained from three separate experiments; at least 100 conidia per condition were analyzed.

**FIGURE 5:**
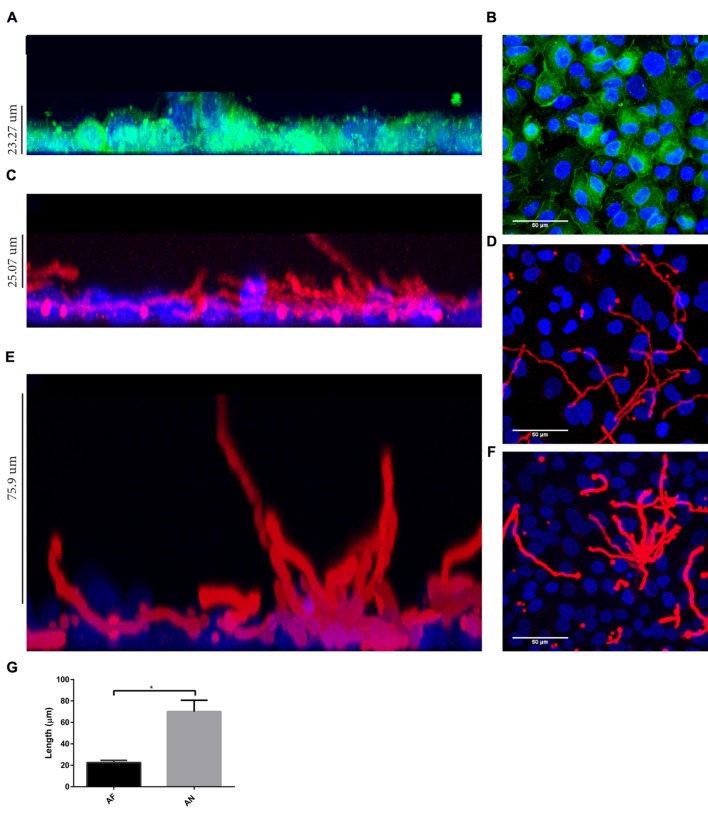
***Aspergillus fumigatus* hyphae grow parallel to A549 cell layer whereas *A. niger* hyphae grow more perpendicularly.** Direction of hyphal growth of *A. fumigatus* and *A. niger* in the presence of A549 cells: **(A)** Z-plane showing thickness of A549 cell layer, nuclei are stained with Hoechst (blue) and cell contour by CellMask^TM^ (green). **(B)** A549 cell layer X/Y-plane. Z-plane **(C,E)** and X/Y-plane **(D,F)** showing *A. fumigatus* **(C,D)** and *A. niger* growth **(E,F)** on A549 (Hoechst stained) cells. **(G)** Hyphal growth of *A. fumigatus* and *A. niger* in the Z-plane. Bars represent standard deviation. ^∗^ Indicates significant difference. Approximately 10 fields per slide from three biological replicas were analyzed.

Interestingly, by using a nystatin protection assay we observed that all internalized conidia from *A. fumigatus* and *A. niger* were viable after 20 h (**Supplementary Figure S3**). As part of the *Aspergillus* cycle of infection, internalized conidia need to escape from cellular endosomes. Germination mediates this step and is essential for dissemination, enabling the fungi to reach new organs. Therefore, the germination time in the absence or presence of epithelial cells was investigated. In cell culture medium, germ tubes appeared after 6 and 5 h for *A. fumigatus* and *A. niger*, respectively. Germination was delayed for 2 h in the presence of A549 cells (**Supplementary Figure S4**). At 10 h after infection as little as 6% of the conidia of *A. fumigatus* had germinated, while at 9 h, 24% of *A. niger* conidia had already germinated (**Figure 4A**). Germtubes also differed in length. *A. fumigatus* hyphae were significantly smaller compared to those of *A. niger* (**Figure 4B**). In addition, hyphae of *A. fumigatus* and *A. niger* showed a difference in growth direction. Hyphae of *A. fumigatus* grew mainly parallel to the A549 epithelial cell layer (reaching maximally 22 μm above the cell layer), while hyphae of *A. niger* grew perpendicular to the cell layer, reaching 70 μm above the epithelial cell monolayer (**Figure 5**; **Figure 5G** for quantification). These observations were not related to differences in growth since hyphae of both strains were grown until a comparable size prior to the analysis (**Figures 5D,F**). However, to prove that these differences are not due to growth, the ratio of hyphae perpendicular to the cells vs. the number of hyphae parallel to the cells was calculated. For *A. fumigatus* this ratio was 1:3 in contrast to *A. niger* which gave a ratio of 2:1.

### Effects of Neutrophils on *A. niger* and *A. fumigatus* Infection

**FIGURE 6:**
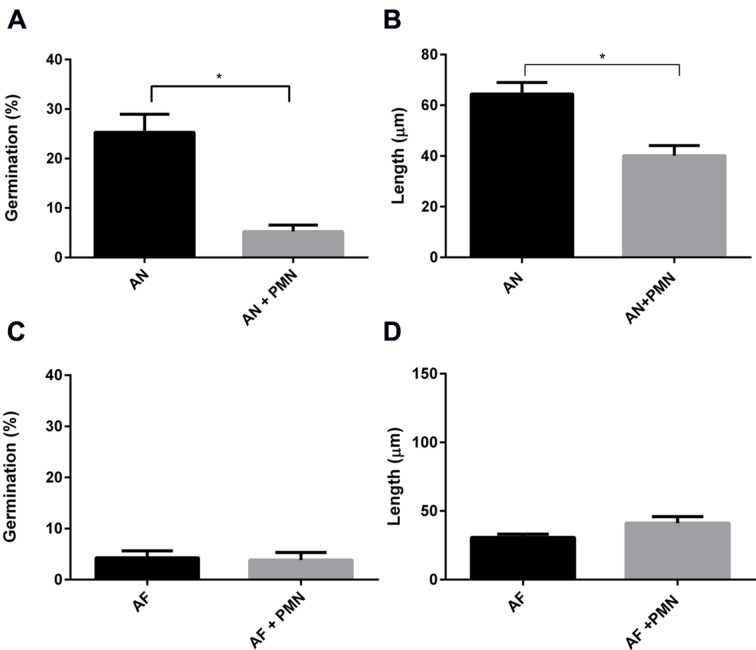
**Polymorphonuclear neutrophils reduce *A. niger* germination and hyphal length at the surface of A549 cells.** *A. fumigatus* and *A. niger* percentage of germination **(A,C)** and hyphal length **(B,D)** in the presence of A549 cells. Bars represent standard error of the mean. ^∗^ Indicate significant differences. Data are obtained from three separate experiments; at least 100 conidia per condition were analyzed.

**FIGURE 7:**
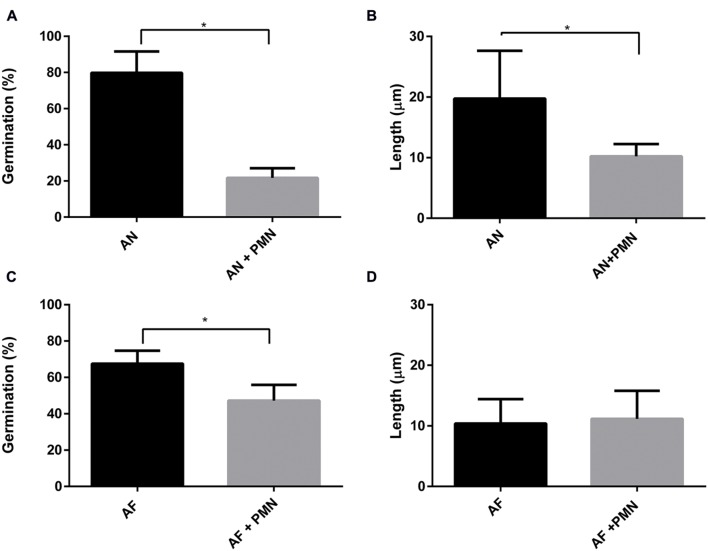
***Aspergillus niger* germination and hyphal length are reduced more effectively by PMNs compared to *A. fumigatus*.** *A. fumigatus* and *A. niger.* **(A,C)** Percentage of germination, **(B,D)** hyphal length. Bars represent standard error of the mean. ^∗^ Indicate significant differences. Data are obtained from three separate experiments; at least 100 conidia per condition were analyzed.

Polymorphonuclear neutrophils are crucial in controlling IA by killing conidia and hyphal structures by means of phagocytosis or by degranulation and NETs formation (McCormick et al., 2010a; Braem et al., 2015). Here, the effect of PMNs was investigated on germination and hyphal growth of *A. fumigatus* and *A. niger*. To this end, PMNs were added to the A549 monolayer that had been challenged with *A. fumigatus* and *A. niger* conidia. PMNs did not inhibit germination of conidia of *A. fumigatus* and the outgrowth of hyphae (**Figures 6C,D**). In contrast, germination of conidia of *A. niger* was reduced by 31% in the presence of PMNs (**Figure 6A**). In addition, the length of *A. niger* hyphae was reduced by 24% (**Figure 6B**). The effect of PMNs on germination and hyphal length was also studied in the absence of epithelial cells. The inhibitory effects of PMNs were stronger in the absence of A549. Germination of *A. niger* conidia was reduced by 73% in the presence of PMNs, whereas hyphal length was reduced by 50% (**Figures 7A,B**). Germination of conidia of *A. fumigatus* was reduced by 31% with no significant effects on hyphal length (**Figures 7C,D**).

## Discussion

Particles with a size <5 μm, such as fungal conidia, can reach the alveoli if they are not expelled through mucociliary clearance or cleared by phagocytic cells roaming the lung. The interaction between conidia, epithelial cells, and phagocytes within the alveoli will determine whether a successful infection can occur. Adherence and internalization of conidia to epithelial cells and subsequent germination and hyphal outgrowth represent the first stages of infection. In this study the interaction of *A. niger* and *A. fumigatus* conidia with A549 cells, representing the lung type II pneumocytes in alveoli, and neutrophils was studied. Results showed that *A. niger* and *A. fumigatus* adhered equally well to A549 cells but the latter conidia were 17% more internalized by A549 cells. This suggests that A549 cells interact differently with *A. niger* and *A. fumigatus*. Indeed, *A. fumigatus* internalization was inhibited by 57% when actin polymerization was disrupted, while internalization was only reduced by 21% in the case of *A. niger*. Inhibition of microtubule formation did not affect either *A. fumigatus* or *A. niger* internalization.

Epithelial-internalized *A. fumigatus* conidia have been suggested to be a source for reinfection since they are not killed and remain latent inside these cells (Wasylnka and Moore, 2003; Amin et al., 2014). In fact, we observed that all internalized conidia were viable after 20 h. Maintained viability of conidia can be explained when conidia-containing-compartments do not fuse with lysosomes, when acidification of phagolysosomes is actively inhibited, or when conidia survive the low pH in the phagolysosomes. Our results indicate that approximately 50% of the conidia are localized inside LAMP-1 labeled compartments within 8 h. This strongly suggests that half of the internalized population will not end up in lysosomes and therefore remain in a less hostile environment. pH sensitive probes indicated that conidia of *A. fumigatus* are located in compartments with a pH of ±6 up to 6 h after infection. In contrast, compartments containing *A. niger* conidia had acidified to a pH of 4 after 4 h. The latter indicates a stronger acidification of phagolysosomes containing *A. niger* conidia. The fact that all conidia of *A. niger* remained viable within A549 cells implies that these conidia survive the acid conditions within phagolysosomes.

The fact that the pH of phagolysosomes is reduced in the presence of *A. niger* conidia but not in the case of *A. fumigatus* may be explained by the types of melanin that are produced by *A. niger* and *A. fumigatus* (Pal et al., 2014). Indeed, conidia of a white strain of *A. niger* showed a similar acidification as the wild-type, whereas white conidia of *A. fumigatus* showed an acidification similar to wild-type *A. niger*. The latter result is in agreement with previous studies showing that the DHN-melanin deficient *A. fumigatus* strain *ΔpksP* failed to inhibit phagosomal acidification (Jahn et al., 2002; Thywissen et al., 2011; Heinekamp et al., 2012; Amin et al., 2014). It should be mentioned, however, that we did not detect acidification of phagolysosomes with LysoSensor^TM^ and that we noticed an unspecific binding of LysoSensor^TM^ to conidia lacking melanine (**Supplementary Figure S2**). The difference in melanin type between *A. niger* and *A. fumigatus* could also explain why *A. fumigatus* conidia are more efficiently internalized. In fact, internalization of *A. fumigatus* conidia in A549 cells is enhanced in the presence of DHN-melanine (Amin et al., 2014).

Germination of *A. niger* and *A. fumigatus* conidia was inhibited by both A549 and PMNs. Germination of both types of conidia was delayed for approximately 2 h at the A549 epithelium. Moreover, the number of conidia of germinated *A. fumigatus* was reduced 13-fold in the presence of A549 cells whereas it was only threefold reduced for *A. niger* conidia (data obtained from **Figures 6** and **7**). Compared to *A. niger*, *A. fumigatus* conidia germinated 20% less and had a twofold decrease in hyphal length in the presence of A549 cells (**Figure 6**). Interestingly, PMNs had a significant effect on *A. niger* germination but not on *A. fumigatus* germination, either in the presence or absence of A549 cells.

Hyphae of *A. niger* and *A. fumigatus* showed a different growth direction in the presence of A549 cells. Most *A. niger* hyphae grew perpendicular to the A549 monolayer, whereas those of *A. fumigatus* grew parallel to the cell layer. The reason for this behavior is not clear. It could be related to stimuli-dependent growth or tropism toward nutrients or oxygen. Hyphal oxytropism has been described for the fungal human pathogen *Candida albicans*, showing elongation toward oxygen-rich environments (Aoki et al., 1998). This may also be the case for aspergilli. They need a minimal oxygen concentration between 0.1 and 0.5% for growth (Hall and Denning, 1994). It has been reported that *A. niger* hyphae respond to electrical fields, chemical compounds, and topographical sensing (Bowen et al., 2007). Little is known about hyphal tropism or contour sensing in *A. fumigatus.* The differences in hyphal orientation may also be due to a stronger interaction of *A. fumigatus* hyphae with epithelial cells as compared to those of *A. niger*.

Internalization by epithelium hides conidia from phagocytic recognition, increasing chances of germination and infection (Wasylnka and Moore, 2003; Lopes Bezerra and Filler, 2004). We hypothesized that *A. niger* hyphae that are more exposed in the medium would render them more susceptible to phagocytic recognition. Indeed, neutrophils reduced germination of *A. niger* conidia in the presence of A549 cells by 20% and hyphal growth by 38%. Reduction was also observed in the absence of the epithelial cells. Decreased hyphal growth was not observed in the case of *A. fumigatus*, when PMN were added (**Figure 7**). Our results support the hypothesis that conidia internalized by epithelial cell hide from neutrophil recognition. However, once they germinate and grow out of these cells they may be attacked. Possibly, parallel growth to the cell layer may reduce neutrophil attack. *A. fumigatus* is known to secrete a complex extracellular matrix composed of mono- and polysaccharides and proteins including antigens and hydrophobins. This matrix is known to function in adherence and makes fungi more resistant to antifungals (Müller et al., 2011). Possibly, *A. niger* fails to produce a similar extracellular matrix, making it more susceptible for immune recognition.

Taken together, our results show differences between *A. niger* and *A. fumigatus* in an *in vitro* model. These observations show a strong difference in the behavior of *A. niger* compared to *A. fumigatus* that could give an advantage to the latter during lung infection. A higher internalization rate and a delay in germination will result in efficient hiding for clearance by the immune system and provides a latent conidia population that can cause an infection. *A. fumigatus* conidia are also less sensitive to PMNs when compared to *A. niger.* The tight contact of *A. fumigatus* with the epithelial surface may help to evade phagocytic recognition. Future studies with other *Aspergillus* species are needed to clarify if the observed advantages are unique for *A. fumigatus* or shared with other species such as *A. terreus* or *A. flavus*.

## Author Contributions

NE, SO, HC, and HH contributed to the design of the work. NE and SO performed the experiments. All authors were involved in the analysis and interpretation of data. NE and SO wrote the manuscript. HW, P-JH, HC, and HH made revisions. All authors approved the version to be published and agreed to be accountable for all aspects of the work.

## Conflict of Interest Statement

The authors declare that the research was conducted in the absence of any commercial or financial relationships that could be construed as a potential conflict of interest.

## Supplementary material

FIGURE S1 **Internalization by A549 cells of *A. fumigatus* conidia (light gray shading) is higher than *A. niger* conidia (black shading) after 2 h. (A)** Association of conidia in time, expressed as the number of conidia per A549 cells. **(B)** Association of conidia after 4 h, expressed as the percentage of the total inoculum. **(C)** Internalization of conidia in time expressed as the number of conidia per A549 cell. **(D)** Internalization of total inoculum after 4 h. Data from four independent experiments from more than 200 conidia per strain.

FIGURE S2 **LysoSensor^TM^ did neither colocalize with *A. fumigatus* nor with *A. niger* but binding of LysoSensor to melanin knockout mutants (white) in the absence of A549 cells was observed. (A)** Conidia of *A. fumigatus* and *A. niger* labeled with red fluorescent protein inside A549 cells 8 h after the challenge. LysoSensor^TM^ (green) labeled acidic compartments. **(B)** Conidia of the white strains of *A. niger* co-localizing in A549 cells with LysoSensor^TM^ 8 h after challenge. **(C)** Conidia of the white strains of *A. fumigatus* and *A. niger* in culture medium bound to LysoSensor^TM^. Data are from one of three representative experiments.

FIGURE S3 ***Aspergillus fumigatus* and *A. niger* conidia survive inside A549 cells.** *A. fumigatus* and *A. niger* inside A549 cells after 3 h and overnight (20 h). Nystatin (Nyst) protection assay was used to kill conidia outside the cells. Data from one of three representative experiments.

FIGURE S4 **Germination of *A. fumigatus* and *A. niger* conidia was 2 h delayed in the presence of A549 cells. (A)** Start of conidial germination in the absence and presence of A549 cells; Arrows indicate germtubes. **(B)** Germination of conidia in the absence of A549 cells after 8 h of growth on DMEM. *A. fumigatus* and *A. niger*. Data are from one of three representative experiments.
