# Supplementary figures and images for: Hide, Keep Quiet, and Keep Low: Properties That Make Aspergillus fumigatus a Successful Lung Pathogen
Source: Front Microbiol. 2016 Apr 6;7:438. doi: 10.3389/fmicb.2016.00438 (PMC4821987; doi:10.3389/fmicb.2016.00438)

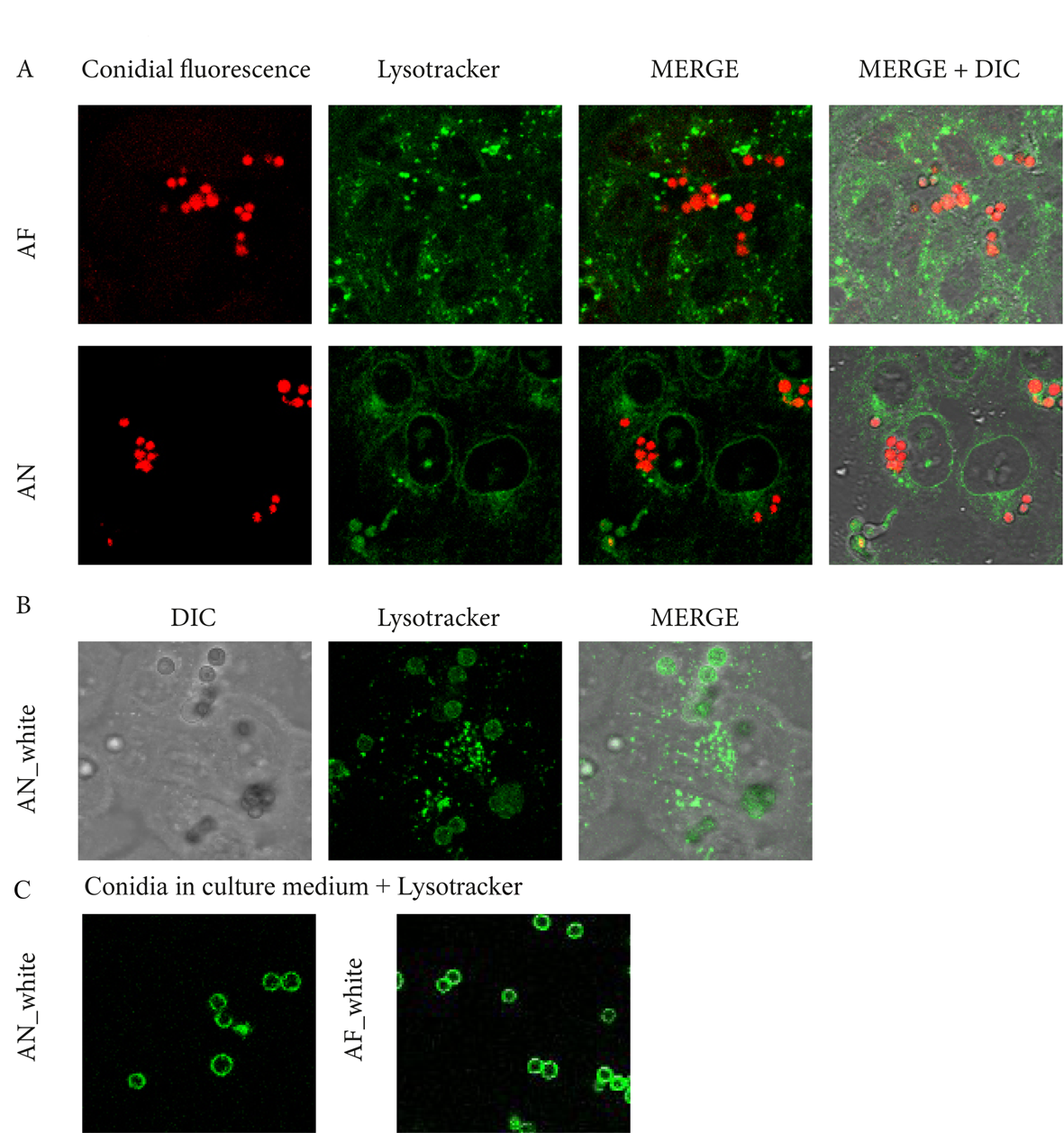

Supplement: FIGURE S2 — LysoSensorTM did neither colocalize with A. fumigatus nor with A. niger but binding of LysoSensor to melanin knockout mutants (white) in the absence of A549 cells was observed. (A) Conidia of A. fumigatus and A. niger labeled with red fluorescent protein inside A549 cells 8 h after the challenge. LysoSensorTM (green) labeled acidic compartments. (B) Conidia of the white strains of A. niger co-localizing in A549 cells with LysoSensorTM 8 h after challenge. (C) Conidia of the white strains of A. fumigatus and A. niger in culture medium bound to LysoSensorTM. Data are from one of three representative experiments. [file Image_2.TIF]

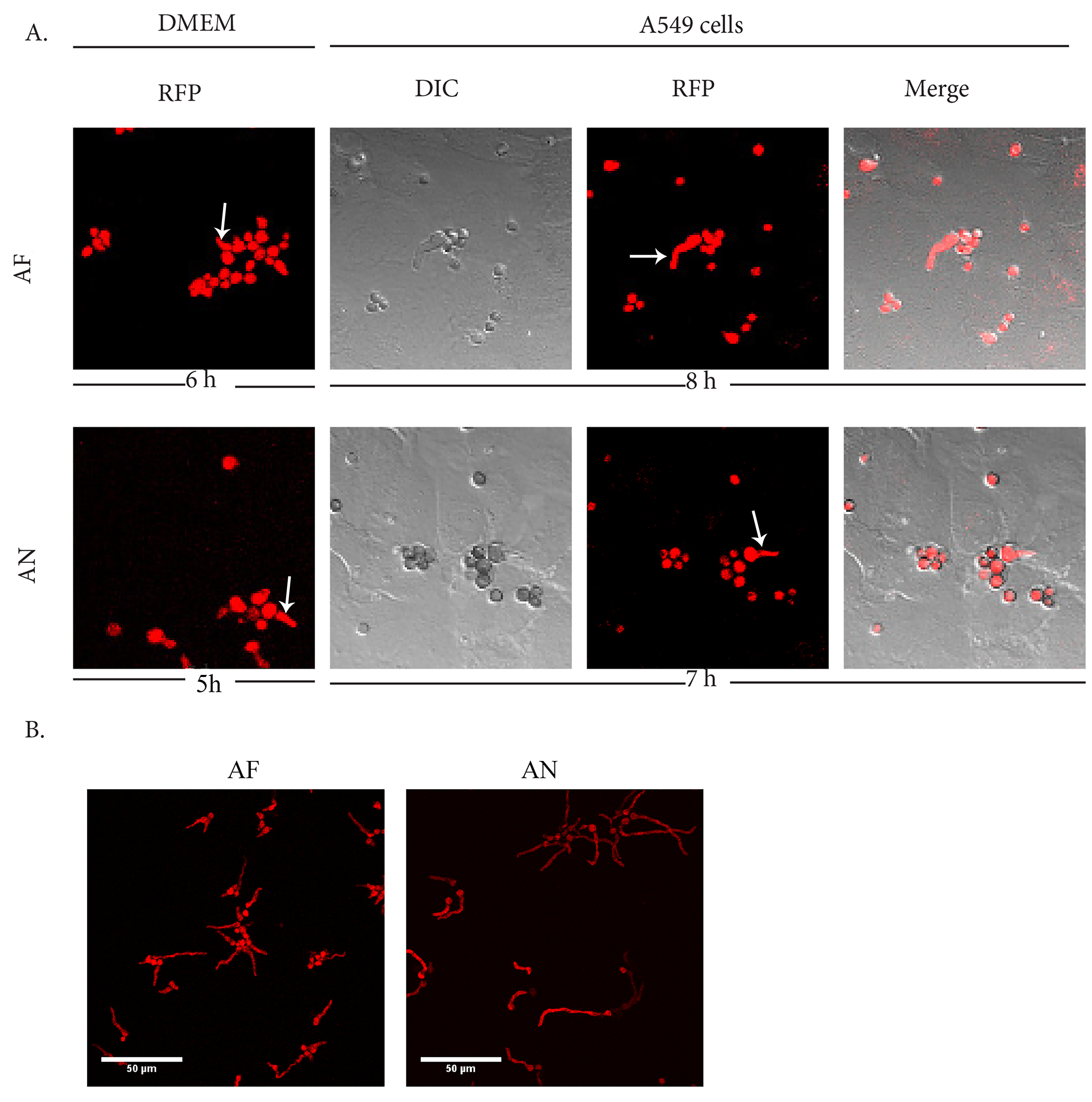

Supplement: FIGURE S4 — Germination of A. fumigatus and A. niger conidia was 2 h delayed in the presence of A549 cells. (A) Start of conidial germination in the absence and presence of A549 cells; Arrows indicate germtubes. (B) Germination of conidia in the absence of A549 cells after 8 h of growth on DMEM. A. fumigatus and A. niger. Data are from one of three representative experiments. [file Image_4.TIF]
